# Investigation of newborn blood metabolomics in varying intrauterine growth conditions

**DOI:** 10.1016/j.jped.2024.07.009

**Published:** 2024-08-21

**Authors:** Shengwen Wang, Xiaofei Lin, Yu Zhou, Xin Yang, Mingming Ou, Linxin Zhang, Yumei Wang, Jing Gao

**Affiliations:** a Huai'an Maternal and Child Health Care Hospital Affiliated to Yangzhou University, Department of Children's Rehabilitation, Jiangsu Province, China; b Huai'an Maternal and Child Health Care Hospital Affiliated to Yangzhou University, Department of Pediatrics, Jiangsu Province, China; c Huai'an Maternal and Child Health Care Hospital Affiliated to Yangzhou University, Neonatal Disease Screening Center, Jiangsu Province, China

**Keywords:** Metabolomics, Small for gestational age, Large for gestational age, Amino acid, Carnitine

## Abstract

**Objectives:**

This study aimed to investigate changes in the blood metabolic profiles of newborns with varying intrauterine growth conditions. Specifically, we analyzed the levels of amino acids, carnitine, and succinylacetone among full-term newborns, including small for gestational age (SGA), appropriate for gestational age (AGA), and large for gestational age (LGA). We aim to identify differential metabolites and metabolic pathways that may offer insights into clinical interventions.

**Methods:**

A total of 5106 full-term newborns were included in the study. Blood samples were obtained from all newborns between 3 and 5 days after birth and analyzed using tandem mass spectrometry to detect blood metabolites. Subsequently, we screened for different metabolites and metabolic pathways among the groups using the MetaboAnalystR package (Version 1.0.1) in R software (R-3.6.0).

**Results:**

The levels of blood amino acids and carnitine metabolism differed significantly among newborns with varying intrauterine growth conditions. Full-term SGA newborns exhibited a decrease in multiple amino acids and an increase in multiple carnitines, while full-term LGA newborns showed an increase in multiple amino acids and acylcarnitines.

**Conclusion:**

Continuous monitoring of the short-term and long-term growth and metabolic status of full-term SGA and LGA newborns is warranted with individualized dietary and nutritional adjustments to promote healthy growth in a timely manner. The findings of this research contribute to the broader understanding of SGA/LGA and shall inform future research on metabolomics, interventions, and long-term outcomes.

## Introduction

The theory of the Developmental Origins of Health and Disease (DOHaD) posits that fundamental life processes undergo changes due to metabolic and nutritional environmental fluctuations during prenatal and early postnatal life.^1^ These changes can lead to an elevated risk of chronic illnesses later in life. Fetal growth and development rely on the balance and regulation of maternal nutrient supply, placental nutrient transportation, genetic factors, and a healthy metabolic environment during pregnancy. After birth, a newborn's metabolism undergoes significant changes as they transition from the maternal environment.^2^ Therefore, understanding the metabolic profile of a newborn and the metabolic changes influenced by various factors during the prenatal and neonatal periods holds paramount importance for nutrition support, growth and development, disease treatment, and prognosis.

Birth weight not only reflects prenatal growth and nutrition but is also closely linked to the risk of perinatal diseases, short- and long-term growth restrictions, obesity, and chronic conditions like hypertension and diabetes in adulthood. According to birth weight standards for infants of different gestational ages, newborns are categorized as small for gestational age (SGA), appropriate for gestational age (AGA), or large for gestational age (LGA). SGA denotes a birth weight below the 10th percentile for infants of the same gestational age and sex, AGA encompasses birth weights between the 10th and 90th percentiles, while LGA signifies birth weights above the 90th percentile.^3^^,^^4^

Both SGA and LGA are common complications of pregnancy with varying incidence rates worldwide.^5^, ^6^, ^7^, ^8^, ^9^, ^10^, ^11^ The adverse intrauterine environment associated with SGA can impede fetal growth and have long-term health impacts, including increased mortality, stunted growth, delayed neural development, and a higher risk of adult diabetes, metabolic syndrome, and cardiovascular diseases.^9^^,^^12^, ^13^, ^14^, ^15^, ^16^ Similarly, the prevalence of LGA varies greatly, ranging from 4.3 % to 22.1 %.^11^ Full-term LGA not only prolongs the delivery process but also increases the risks of cesarean section, postpartum hemorrhage, birth injury, fetal distress, shoulder dystocia, brachial plexus injury, and clavicle fracture. Additionally, due to excessive weight in the uterus, LGA increases the risks of overweight, obesity, and diabetes in the long run.^17^, ^18^, ^19^

Metabolomics, a rapidly emerging science following genomics and proteomics, enables the study of an individual's metabolic state at a specific moment. It quantitatively and qualitatively assesses metabolites in biological specimens, offering insight into an organism's physiological and pathological conditions. This approach allows us to explore the growth and development processes of fetuses and newborns, as well as the origins and development of long-term diseases.

Despite the focus on the incidence, etiology, and short- and long-term health impacts of SGA and LGA newborns by scholars worldwide, limited research has applied metabolomics analysis to these groups. However, studies have shown significant differences in amino acid and carnitine levels between SGA/LGA and AGA newborns. Nonetheless, comprehensive studies on blood amino acid and carnitine metabolism, as well as their differential metabolites and metabolic mechanisms in full-term SGA/LGA newborns, particularly full-term LGA newborns, are scarce.

Hence, this study employs tandem mass spectrometry to analyze the blood amino acid and carnitine indices of full-term newborns from a large-scale birth population. The aim is to identify differential metabolites and metabolic pathways in the blood of full-term SGA, LGA, and AGA newborns, laying the groundwork for improving personalized nutritional solutions in clinical settings. This research could significantly enhance the near- and long-term nutritional metabolism of SGA and LGA newborns and reduce the risk of adverse outcomes.

## Materials and methods

### Study subjects

**Figure 1:**
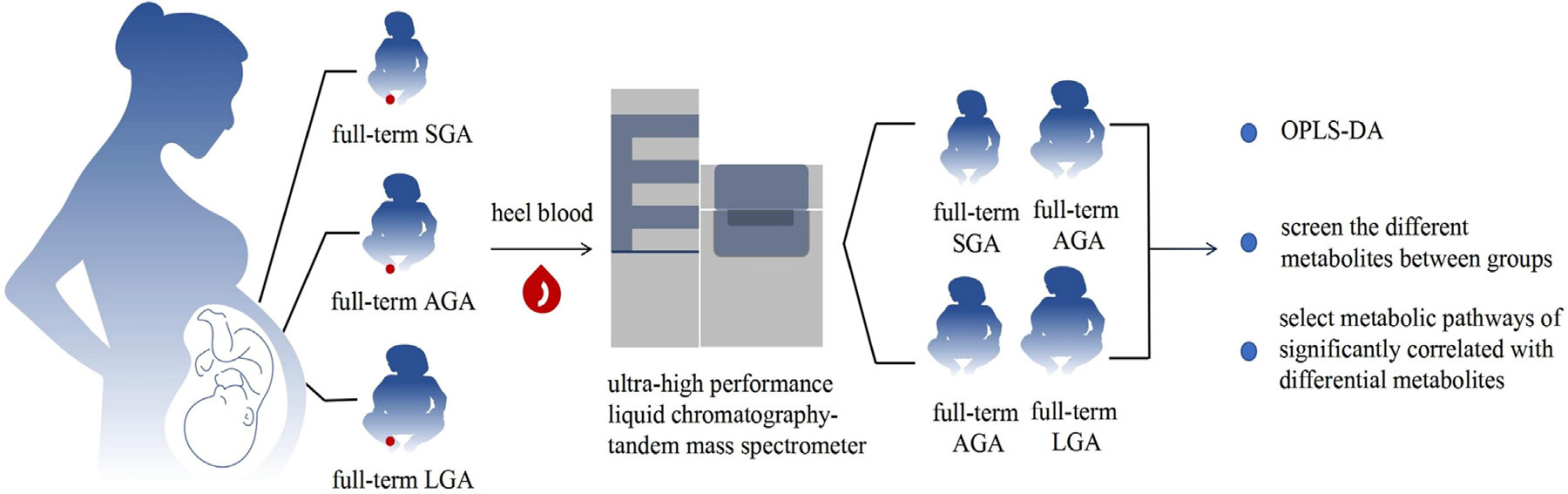
Flowchart.

The study included 5106 full-term newborns delivered between January and March 2022 in various midwifery institutions in Huai'an City. Participants were categorized into three groups based on normal weight standards for infants of different gestational ages: SGA, AGA, and LGA (Figure 1).^3^^,^^4^

Inclusion criteria: (1) the gestational age is between 37 and 42 weeks; (2) singleton birth; (3) good condition after birth, with no congenital malformations, resuscitation or rescue history, severe congenital heart disease, asphyxia, abnormal blood glucose level, etc.; (4) collection of heel blood three to five days after birth; (5) complete medical records.

Exclusion criteria: (1) mother with severe internal or surgical diseases, malnutrition, severe endocrine and metabolic diseases, etc.; (2) diagnosed with inherited metabolic diseases. This study has been approved by the Ethics Committee of Huai'an Maternal and Child Health Care Hospital (No. 2021034), and the newborn guardians have given their informed consent and signature.

### Research methods

#### Sample collection

All newborns were fully breastfed after birth. Heel blood samples were collected by trained professionals between 3 and 5 days post-birth. The collection process adhered to "Technical Specifications for Blood Collection for Neonatal Disease Screening." Blood samples were carefully applied to filter paper (Scheicherand and Schuell 903#) to create dried blood spots. Each blood spot had a diameter of more than 8 mm. After sample application, the filter papers were left to air dry naturally. Subsequently, the dried blood spot samples were placed in transparent sealed bags and stored in a −20 °C freezer for subsequent testing.

#### Testing indicators

A total of 43 metabolic indicators were tested, including (1) 11 amino acids: TYR, ALA, ARG, ORN, CIT, GLY, VAL, LEU, MET, PHE and PRO; (2) 31 carnitines: C0, C2, C3, C3DC+C4OH, C4, C4DC+C5OH, C5, C5DC+C6OH, C5:1, C6, C6DC, C8, C8:1, C10, C10:1, C10:2, C12, C12:1, C14, C14:1, C14:2, C14OH, C16, C16:1, C16OH, C16:1OH, C18, C18:1, C18:2, C18OH and C18:1OH; (3) SA.

#### Instruments and reagents

The following instruments and reagents were used: the Puncher^9^ fully automatic punching machine produced by Finland Perkin Elmer Company, single-channel and eight-channel quantitative dispensers and assistant suction pumps produced by Eppendorf Company of Germany, 10 ml and 50 ml glass pipettes, Hangzhou Oshen MB100-4A incubator shaker, Sumi KQ-100E ultrasonic cleaner, nitrogen and argon gases, and the TQ-D ultra-high performance liquid chromatography-tandem mass spectrometer produced by the American Waters Company. The non-derivatization method was applied to test multiple amino acids, carnitines and succinylacetone using the Perkin Elmer assay kits.

#### Sample preparation

Using an automatic punching machine, a 3.2 mm dry blood spot filter paper was taken and placed in a 96-well U-shaped reaction plate. Two wells were set for high and low concentration quality control blood spots, respectively. 100μl of prepared internal standard mixed solution and extraction solution were added. The plate was sealed with a transparent film and placed in a 45 °C constant temperature oscillator for 45 min. After cooling, 90μl was transferred to a 96-well V-shaped measurement plate and left at room temperature for 2 h before detection.

### Tandem mass spectrometry detection

Mass spectrometry ionizes the sample into charged ions, separates them based on different mass-to-charge ratios, and analyzes the structure and composition of the sample while also determining its concentration. The tandem mass spectrometer uses electrospray ionization technology with an ionization voltage of 3.5 kV and a desolvation gas temperature of 350 °C during detection.

### Quality control

The low concentration and high concentration quality control products provided with the test kit were used for each batch of experiments, and quality control analysis was performed on the data of each batch of experiments. The laboratory participates in the inter-laboratory quality assessment organized by the National Clinical Laboratory Center of the National Health Commission twice a year.

### Data analysis

Data analysis was conducted using SPSS 26.0 statistical software. The presentation of results is as follows: Count data were expressed as the number of cases, and intergroup comparisons were made using the χ² test. Normally distributed measurement data were presented as mean ± standard deviation. Intergroup comparisons were performed using one-way analysis of variance (ANOVA). Statistical significance was defined as *p* < 0.05.

Metabolomic analysis: Orthogonal partial least squares discriminant analysis (OPLS-DA) was performed using R (R-3.6.0) MetaboAnalystR package (Version 1.0.1) for differential analysis. The variable importance in projection (VIP) values obtained from the model and *t*-tests were used for differential analysis. Differential metabolites were screened with VIP>1 and P-value<0.05, and a volcano plot of differential metabolites was produced using the ggplot2 package. Then, MetaboAnalystR package (Version 1.0.1) was used for KEGG metabolic pathway enrichment analysis of differential metabolites.

## Results

### General information of study subjects

Of these newborns, 2592 were male, and 2514 were female. The full-term SGA group comprised 173 cases with 92 males and 81 females. The AGA group included 4486 cases with 2268 males and 2218 females. The LGA group consisted of 447 cases with 232 males and 215 females. Importantly, there were no statistically significant differences in gender and gestational age among the three groups (*p* > 0.05), while there were statistically significant differences in birth weight among the three groups (*p* < 0.05).

### Metabolomic analysis of blood from term SGA and AGA newborns

#### OPLS-DA analysis

**Figure 2:**
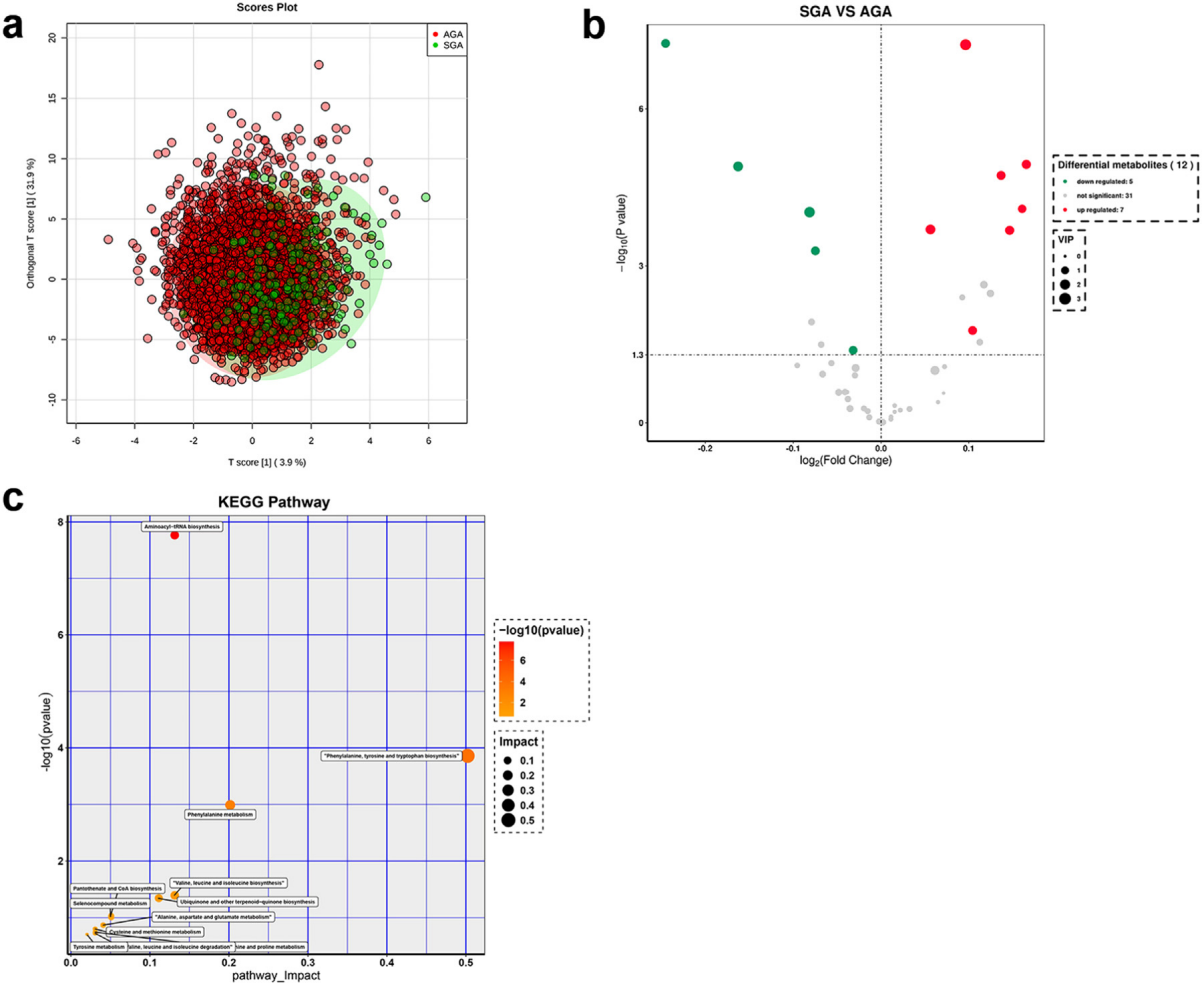
Metabolomic analysis of blood from term SGA and AGA newborns. (a) Clustering chart of orthogonal partial least squares discriminant analysis (OPLS-DA) for term SGA and AGA groups. (b) Volcano plot of differential metabolites between term SGA and AGA groups. (c) The pathway analysis of the SGA and AGA groups at term (POS-Pathway Analysis). The y-axis represents the value of -log10 (P-value) and the x-axis represents the Rich factor. SGA: small for gestational age; AGA: appropriate for gestational age.

The clustering chart (Figure 2a) resulting from the OPLS-DA analysis demonstrated a distinct clustering trend between the metabolic profiles of term SGA and AGA newborns, underscoring differences in the metabolic patterns of these two groups of newborns.

#### Differential metabolite screening between term SGA and AGA newborns

Differential metabolite screening criteria (VIP > 1 and p-value < 0.05) were applied, and the volcano plot (Figure 2b) revealed that when compared with term AGA newborns, seven metabolites in the blood of term SGA newborns were upregulated (C0, C10:1, C10:2, C14:2, C18:2, ALA, and PRO) while five metabolites were downregulated (VAL, MET, PHE, TYR, and C3), all of which can be found in the Human Metabolome Database (HMDB) (Supplementary Table 1).

#### Pathway analysis of differential metabolites

Key pathways displaying the most significant correlation with differential metabolites were identified through enrichment analysis and topological analysis, as illustrated in the bubble plot (Figure 2c). Based on an impact factor greater than 0 in topological analysis and the biological functions of metabolic pathways, 12 significantly enriched metabolic pathways were identified from differential metabolites. These pathways encompassed aminoacyl-tRNA biosynthesis, phenylalanine, tyrosine and tryptophan biosynthesis, phenylalanine metabolism, valine, leucine, and isoleucine biosynthesis, ubiquinone and other terpenoid-quinone biosynthesis, pantothenate and CoA biosynthesis, selenocompound metabolism, alanine, aspartate, and glutamate metabolism, cysteine and methionine metabolism, arginine and proline metabolism, valine, leucine, and isoleucine degradation, and tyrosine metabolism.

### Metabolomic analysis of blood in LGA and AGA newborns at term

#### OPLS-DA analysis

**Figure 3:**
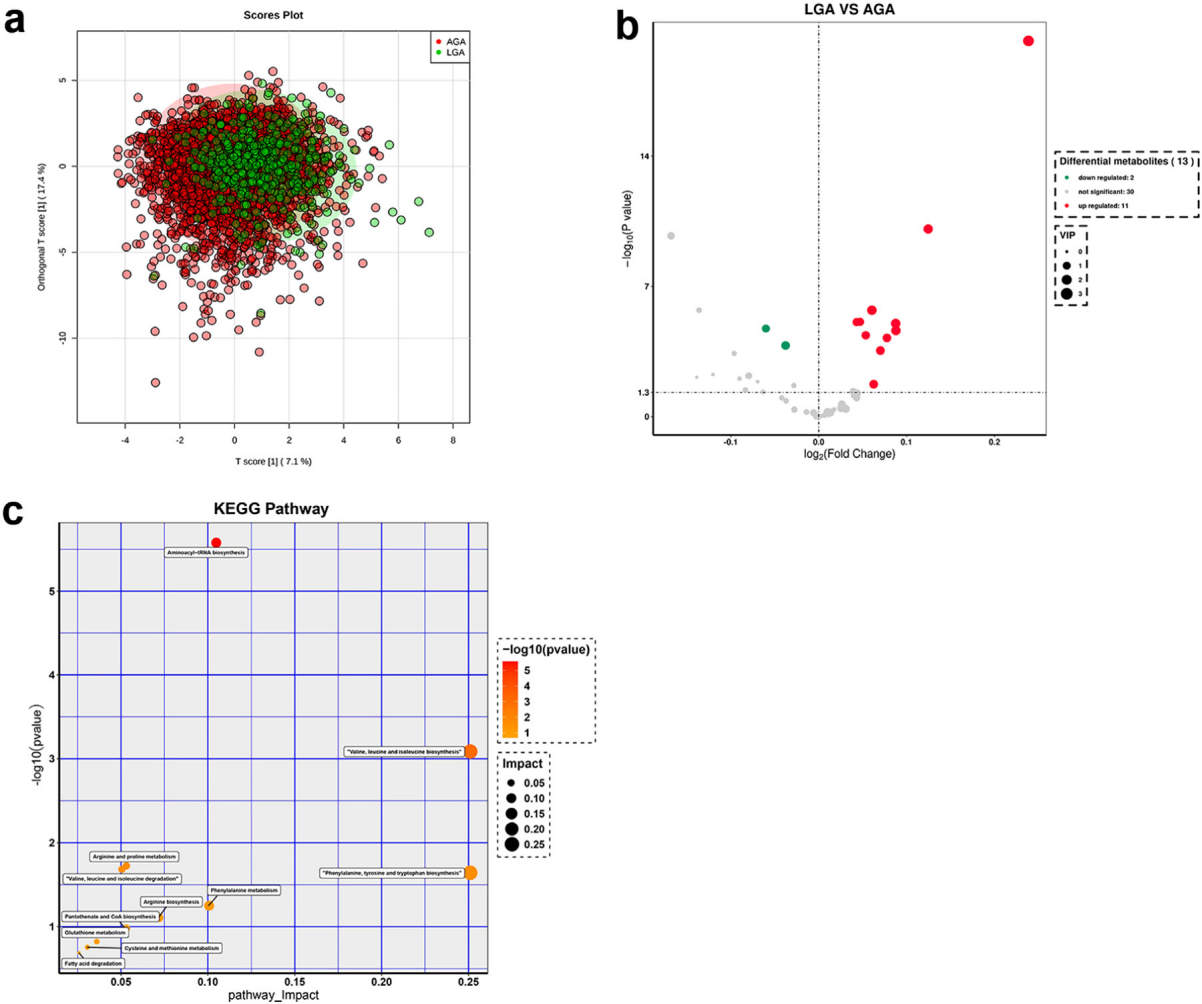
Metabolomic analysis of blood in LGA and AGA newborns at term. (a) The orthogonal partial least squares discriminant analysis clustering plot of term LGA and AGA newborns. (b) The volcano plot of the LGA and AGA groups at term. (c) The pathway analysis of the LGA and AGA groups at term (POS-Pathway Analysis). The y-axis represents the value of -log10 (P-value), and the x-axis represents the Rich factor. AGA: appropriate for gestational age; LGA: large for gestational age.

The clustering plot (Figure 3a) obtained from OPLS-DA analysis reveals a significant clustering trend in the metabolic profiles of term LGA and AGA newborns, indicating that the metabolic patterns of the two groups of newborns were different.

#### Screening of differential metabolites in blood of LGA and AGA newborns at term

After screening for differential metabolites using a VIP > 1 and P-value < 0.05 threshold, the volcano plot (Figure 3b) shows that 11 metabolites were upregulated in the blood of term LGA newborns compared to term AGA newborns: C2, C3, C4DC+C5OH, C5, C5DC+C6OH, C16, C18:1, PHE, MET, LEU, and VAL. Two metabolites were downregulated in LGA newborns, namely PRO and ORN (Supplementary Table 2). All of these differential metabolites can be searched and verified in HMDB.

#### Pathway analysis related to differential metabolites

Key pathways significantly associated with differential metabolites were identified through a comprehensive analysis of enrichment and topological assessments, and the results were graphically represented in a bubble chart (Figure 3c). This study employed a rigorous criterion, considering the impact factor (impact > 0) in topological analysis and the biological implications of metabolic pathways to select 11 significantly enriched metabolic pathways relevant to differential metabolites. These pathways encompassed aminoacyl-tRNA biosynthesis, valine, leucine, and isoleucine biosynthesis, arginine and proline metabolism, valine, leucine, and isoleucine degradation, phenylalanine, tyrosine, and tryptophan biosynthesis, phenylalanine metabolism, arginine biosynthesis, pantothenate and CoA biosynthesis, glutathione metabolism, cysteine and methionine metabolism, and fatty acid degradation.

## Discussion

Nutrition support during the fetal and neonatal stages is crucial for growth, development, and overall metabolism, exerting a profound influence on long-term health outcomes.^20^ Previous research has shown an association between reduced tyrosine levels and fetal growth restriction.^21^ In this study, blood tyrosine levels in full-term SGA newborns were lower than those in full-term AGA newborns, diverging from the findings of Liu et al.^22^ This discrepancy may result from differences in sample measurement methods, collection timing, and sample size. Existing literature also reports lower levels of phenylalanine, leucine, and other amino acids in the umbilical cord blood of SGA infants compared to AGA infants.^23^ Consistent with previous research, this study observed a substantial reduction in phenylalanine levels in the blood of full-term SGA newborns, possibly due to reduced nutrient transport through the placenta during late pregnancy for SGA mothers. On the other hand, consistent with our findings, other studies have indicated increased blood phenylalanine levels in full-term LGA newborns compared to AGA newborns.^24^ It's worth noting that phenylalanine and other aromatic amino acids are metabolic factors linked to metabolic syndrome.^25^ This observation aligns with the phenomenon that LGA newborns tend to experience long-term risks of overweight, obesity, metabolic syndrome, and diabetes.

Previous studies have reported a significant increase in leucine levels in the blood of full-term LGA newborns, whereas the valine levels in the blood of full-term SGA newborns were lower compared to full-term AGA newborns.^26^ The above research is consistent with our findings and may be linked to factors such as inadequate or excessive maternal nutrition intake during pregnancy and placental transport. Current research highlights a correlation between branched-chain amino acids and obesity. These amino acids can serve as predictive markers for insulin resistance, type 2 diabetes, obesity, and cardiovascular disease.^25^^,^^27^ Our study found a significant increase in branched-chain amino acids in the blood of LGA newborns, indicating an increased risk of developing metabolic syndrome in the long term.

In the context of this study, it was observed that in comparison to AGA newborns, full-term SGA newborns exhibited a significant decrease in C3 levels coupled with significantly elevated levels of C0, C10:1, C10:2, C14:2, and C18:2. These findings align with previous research showing increased total and free carnitine levels in SGA groups.^26^ Another retrospective cohort study involving 361 full-term newborns also reported increased levels of C0 and various acylcarnitines in the full-term SGA group, along with a significant decrease in C3 levels.^28^

Healthy newborns primarily rely on glucose and amino acid metabolism for intrauterine energy. However, SGA fetuses, due to inadequate glucose and amino acid intake in utero, must rely more on their own fat reserves, especially medium-chain and long-chain fatty acids, for energy. This compensation likely leads to increased C0 and various medium- and long-chain acylcarnitines in SGA newborns. The substantial decrease in C3 levels in SGA newborns may be related to the reduced levels of branched-chain amino acids, particularly valine, as C3 is a product of mitochondrial metabolism of branched-chain amino acids, especially valine and isoleucine.^29^

Previous studies have reported increased concentrations of short-chain and long-chain acylcarnitines in the LGA group, particularly C2 and C3, which aligns with our findings.^30^ Additional research outcomes have also indicated a significant increase in C3 levels in full-term LGA newborns and a substantial rise in total carnitine levels in severe full-term LGA cases.^26^^,^^28^ However, research into the metabolic environment of term LGA newborns remains limited. The elevated levels of various short- and long-chain acylcarnitines in LGA newborns in this study are likely associated with factors such as maternal obesity prior to pregnancy, excessive nutrient intake during pregnancy, and excessive weight gain during pregnancy. The increase in valine and leucine levels in LGA newborns may further contribute to an increase in their metabolic product, C3.

This study revealed significant variations in blood amino acid and carnitine levels among newborns with differing intrauterine growth statuses. For term SGA newborns, early and appropriate supplementation of aromatic amino acids like tyrosine and phenylalanine, as well as branched-chain amino acids such as valine, is crucial for growth and development. Long-term follow-up is essential to determine the required extent of amino acid supplementation for SGA newborns, aiming to meet their catch-up growth demands while mitigating the risk of excessive intake that may lead to metabolic syndrome and other ailments.

The heightened levels of various carnitines in SGA newborns, attributed to excessive fat breakdown due to amino acid and glucose deficiency, highlight the importance of timely augmenting glucose and amino acid intake. This is critical in averting damage from excessive fatty acid consumption and facilitating catch-up growth. LGA newborns tend to have elevated levels of numerous amino acids and carnitines, which may be associated with excessive nutrient intake, heightened weight gain during pregnancy, maternal obesity, and gestational diabetes.

However, there are still limitations in this study. The study design did not include other clinical characteristics of the participants, such as placental issues, genetics, TORCH infections, maternal factors, diabetes, and idiopathic conditions. Furthermore, the present study only identified some metabolites in newborns without conducting detailed mechanistic or animal model testing. Moving forward, we plan to collaborate with obstetrics, ultrasound departments, genetic centers, and neonatology departments to establish a multidisciplinary treatment model. This collaboration will focus on studying the potential pathological factors that affect fetal and neonatal growth, development, and metabolic status, as well as conducting detailed mechanistic and animal model testing, thereby aiding in the formulation of potential interventional strategies in personalized medicine. We intend to focus our attention on these newborns as key subjects for pediatric care, conducting long-term follow-up management to ensure their well-being.

Continuous monitoring of the short-term and long-term growth and metabolic status of full-term SGA and LGA newborns is vital, along with implementing timely, tailored dietary and nutritional adjustments to foster children's development. The findings of this research contribute to the broader understanding of SGA/LGA and shall inform future research on metabolomics, interventions, and long-term outcomes.

## Funding

This research was funded by Natural Science Research Program Project of Huai'an Science and Technology Bureau (Grant No. HAB201937). The Science and Technology Plan Special Fund of Jiangsu Province (Basic Research Natural Science Foundation, Grant No. BK20230292).

## Institutional review board statement

The study was conducted in accordance with the Declaration of Helsinki and approved by the Ethics Committee of Huai'an Maternal and Child Health Care Hospital (approval No. 2021034).

## Informed consent statement

Informed consent was obtained from all subjects involved in the study.

## Data availability

The data presented in this study are available on request from the corresponding author. The data are not publicly available due to our laboratory's policies.

## CRediT authorship contribution statement

**Shengwen Wang:** Conceptualization, Data curation, Investigation, Methodology, Resources, Writing – original draft, Writing – review & editing. **Xiaofei Lin:** Conceptualization, Writing – original draft, Writing – review & editing. **Yu Zhou:** Data curation, Investigation, Methodology, Funding acquisition, Writing – review & editing. **Xin Yang:** Data curation, Investigation, Methodology, Validation. **Mingming Ou:** Formal analysis, Methodology, Software, Supervision, Validation. **Linxin Zhang:** Conceptualization, Writing – original draft, Writing – review & editing. **Yumei Wang:** Data curation, Funding acquisition, Project administration, Resources, Supervision, Writing – original draft, Writing – review & editing. **Jing Gao:** Data curation, Project administration, Resources, Supervision, Writing – original draft, Writing – review & editing.

## Conflicts of interest

The authors declare no conflict of interest.

## Appendix Supplementary materials

Image, application 1
