# Supplementary material for: Investigation of newborn blood metabolomics in varying intrauterine growth conditions
Source: J Pediatr (Rio J). 2024 Aug 21;101(1):74–81. doi: 10.1016/j.jped.2024.07.009 (PMC11763542; doi:10.1016/j.jped.2024.07.009)
Supplement: Supplementary file 1 [file mmc1.docx]

JPED-D-23-00527 – Supplementary Material

**Supplementary Table 1** Comparison of major differential metabolites in the blood of term SGA and AGA newborns.

| N | MS name | HMDB | Mean AGA(μmol/L) | Mean SGA(μmol/L) | VIP | P-value | FOLD CHANGE |
| --- | --- | --- | --- | --- | --- | --- | --- |
| 1 | alanine | HMDB0000161 | 184.4655489 | 197.2163502 | 2.486314575 | 5.95E-08 | 1.0691 |
| 2 | methionine | HMDB0000696 | 17.370243 | 16.49238106 | 1.340651231 | 0.00051707 | 0.94946 |
| 3 | phenylalanine | HMDB0000159 | 33.63510981 | 31.78921073 | 2.230729952 | 9.41E-05 | 0.94512 |
| 4 | proline | HMDB0000162 | 121.588211 | 126.4280709 | 1.858695442 | 0.00020154 | 1.0398 |
| 5 | tyrosine | HMDB0000158 | 69.81038657 | 62.36258539 | 1.85949275 | 1.26E-05 | 0.89331 |
| 6 | valine | HMDB0000883 | 89.89763375 | 87.94290435 | 1.289982598 | 0.040988 | 0.97826 |
| 7 | carnitine | HMDB0000062 | 13.25897907 | 14.57638856 | 1.419732247 | 1.86E-05 | 1.0994 |
| 8 | propionylcarnitine | HMDB0000824 | 1.16301714 | 0.981180321 | 1.362513699 | 5.62E-08 | 0.84365 |
| 9 | decenoylcarnitine | HMDB0013205 | 0.04579315 | 0.051359568 | 1.521686228 | 1.15E-05 | 1.1216 |
| 10 | decadienoylcarnitine | HMDB0240751 | 0.008903745 | 0.00957178 | 1.361539445 | 0.017233 | 1.075 |
| 11 | tetradecadienoylcarnitine | HMDB0258882 | 0.012390461 | 0.013851256 | 1.184215716 | 8.14E-05 | 1.1179 |
| 12 | octadecadienoylcarnitine | HMDB0240780 | 0.168806026 | 0.186841118 | 1.526698335 | 0.00020976 | 1.1068 |

N, number; MS name, substance matching name; VIP, VIP value from OPLS-DA model; P-VALUE, P value from t-test; FOLD_CHANGE, Quantitative ratio of the two experimental substances; HMDB, Index of the substance in the HMDB database.

**Supplementary Table 2** Comparison of major differential metabolites in the blood of term LGA and AGA newborns.

| N | MS name | HMDB | Mean AGA(μmol/L) | Mean LGA(μmol/L) | VIP | P-value | FOLD CHANGE |
| --- | --- | --- | --- | --- | --- | --- | --- |
| 1 | ornithine | HMDB0000214 | 66.88916622 | 64.15723864 | 1.014830901 | 1.86E-05 | 0.95916 |
| 2 | proline | HMDB0000162 | 121.588211 | 118.4447641 | 1.307598142 | 0.00014956 | 0.97415 |
| 3 | valine | HMDB0000883 | 89.89763375 | 92.6178379 | 1.110952205 | 8.33E-06 | 1.0303 |
| 4 | leucine | HMDB0000687 | 105.3521372 | 108.8482428 | 1.091800417 | 8.07E-06 | 1.0332 |
| 5 | methionine | HMDB0000696 | 17.370243 | 18.02612703 | 1.208039553 | 4.30E-05 | 1.0378 |
| 6 | phenylalanine | HMDB0000159 | 33.63510981 | 35.07344954 | 1.799565301 | 1.93E-06 | 1.0428 |
| 7 | hexadecanoylcarnitine | HMDB0000222 | 1.576654077 | 1.646509169 | 1.354094414 | 0.018214 | 1.0443 |
| 8 | methylmalonyl / 3-hydroxy-isovalerylcarnitine | HMDB0013133 | 0.132956196 | 0.139594215 | 1.426374463 | 0.00028323 | 1.0499 |
| 9 | glutarylcarnitine/  3-hydroxy-hexanoylcarnitine | HMDB0013130 | 0.063136467 | 0.066624802 | 1.273121126 | 5.91E-05 | 1.0553 |
| 10 | octadecenoylcarnitine | HMDB0255949 | 0.804835697 | 0.855200567 | 1.772640916 | 9.88E-06 | 1.0626 |
| 11 | acetylcarnitine | HMDB0000201 | 12.26081425 | 13.03131799 | 1.753656598 | 2.37E-05 | 1.0628 |
| 12 | isovalerylcarnitine | HMDB0000688 | 0.063523669 | 0.069252905 | 1.585434728 | 8.28E-11 | 1.0902 |
| 13 | propionylcarnitine | HMDB0000824 | 1.16301714 | 1.372332365 | 2.441788765 | 6.43E-21 | 1.18 |

N, number; MS name, the matched name of the substance; VIP, the VIP value from the OPLS-DA model; P-value, the P-value from t-test; FOLD_CHANGE, the ratio of quantitative measurement between the two groups; HMDB, the index of the substance in the HMDB database.
